# QTL mapping and transcriptome analysis of sugar content during fruit ripening of *Pyrus pyrifolia*

**DOI:** 10.3389/fpls.2023.1137104

**Published:** 2023-03-06

**Authors:** Shuang Jiang, Shuigen Li, Jun Luo, Xiaoqing Wang, Chunhui Shi

**Affiliations:** Forestry and Pomology Research Institute, Shanghai Key Lab of Protected Horticultural Technology, Shanghai Academy of Agricultural Sciences, Shanghai, China

**Keywords:** sugar content, fruit ripening, QTL mapping, transcript, gene regulation

## Abstract

Sugar content is an important trait of fruits. The genetic background of fruits can affect their sugar content in different cultivars. The quantitative trait loci and genes related to sugar content during fruit ripening remain unclear. In this study, we performed quantitative trait locus (QTL) mapping of sugar content. Two QTLs (qSugar-LG6-Chr7 and qSugar-LG12-Chr3) were identified based on their total sugar contents. A total of 577 and 519 genes were annotated around these two QTL loci. The contents of fructose, sorbitol, glucose, and sucrose were measured at six time points in four cultivars before fruit maturation, including two sweet cultivars (“Zaoshengxinshui” and “ZQ65”) and two general cultivars (“Qiushui” and “ZQ82”). In sweet cultivars, sucrose and fructose accumulate substantially, and sorbitol content decreases significantly during fruit ripening. A transcriptome analysis identified 125 upregulated and 222 downregulated differentially expressed genes (DEGs) in sweet cultivars. Two sucrose transport genes (*PpSUT*, LOC103964096, and LOC103940043) were negatively correlated with sugar content. A weighted gene co-expression network analysis showed that two genes, sorbitol dehydrogenase (*PpSDH*, LOC103960512 and LOC103960513), around the locus of qSugar-LG6-Chr7 were negatively co-expressed with the total sugar content, which was downregulated in the sweet cultivars. *PpSDH* and *PpSUT* may play important roles in regulating sugar content during pear ripening. Transcriptome analysis also revealed that some DEGs were related to sugars (*PpS6PDH* and *ATP-PpPFK*), hormones (*PpARG7*), and transcription factors (*PpEMB1444*, *PpCYP734A1*, and *PpWRKY50*). In conclusion, this study provides new insights into the molecular mechanisms associated with sugar content in the fruit ripening of *Pyrus pyrifolia*.

## Introduction

Fruit ripening is a complex process, and one of its key indicators is total sugar content (Duran-Soria et al., 2020). The sweetness of fruits is a highly important quality that is also strongly associated with commercial value. Genetic background, environmental factors, chemicals, and cultivation techniques regulate the total sugar content during fruit ripening. For example, the total sugar content differed between 9.63% and 29.47% in 178 plum cultivars (Sahamishirazi et al., 2017). The fruits of early maturing cultivars sweeten earlier than those of late-maturing cultivars (Khan, 2016). Drought stress can enhance carbohydrate accumulation (Wu et al., 2017). Fruits are generally sweeter in hot and dry years than in normal years (Liu et al., 2013). Spraying gibberellin on pear fruit can also produce sweet fruit (Li et al., 2015). Organic and foliar fertilizers can increase the sweetness of fruit (del Amor, 2007). Currently, selecting cultivars with high sugar contents is desirable in pear breeding because consumers generally prefer sweet pears.

There are four types of sugars in a pear fruit: sucrose, glucose, fructose, and sorbitol (Pilando and Wrolstad, 1992). The sweetness of the fruit is controlled by the total sugar content and percentage of different individual sugars. Sucrose and sorbitol are the main sugars for carbohydrate transport from source to sink in pears (Wang et al., 1999; Aoki et al., 2012). Several QTLs that are related to sugar content have been identified. A genome-wide association study identified sucrose and glucose contents on chromosome 7 in Japanese pears (Nishio et al., 2021). An acid invertase gene *PpAIV3* was detected within QTL intervals on chromosome 7. Another report found that the total soluble solid content-related QTL was located in chromosomes 4 and 8 in Japanese pears (Yamamoto et al., 2014). Two QTLs were located in Linkage Group 1 (LG 1) and LG 7 based on the sucrose, fructose, and glucose contents in Japanese pears (Nishio et al., 2018). In Chinese pears, three QTLs for soluble solid content were located in LG02, LG05, and LG06 (Zhang et al., 2013). Moreover, two reports identified QTLs for soluble solid content in LG5, LG10, and LG15 (Wu et al., 2014; Qin et al., 2022). The differing QTLs implied that the sugar content was determined by multiple loci.

Several genes participate in the transportation and synthesis of sugars. Seven gene families are related to sugar transportation, including the sugar transporter protein, sugar promoter protein, polyol/monosaccharide transporter, inositol transporter, plastidic glucose transporter, tonoplastmono saccharide transporter, and vacuolar glucose transporter families (Zheng et al., 2014; Khanbo et al., 2021). Invertase is widely expressed in microorganisms, animals, and plants; it can irreversibly hydrolyze sucrose into fructose and glucose and strongly influence the accumulation of sugar in fruits. Based on the optimal pH value, solubility, and subcellular location, invertases can be divided into three types: vacuolar (soluble acid), cytoplasmic (soluble alkaline), and cell wall-bound invertases (Kulshrestha et al., 2013). Sucrose synthase is a key enzyme that promotes the entry of sucrose into various metabolic pathways within fruits (Koch, 2004). There are two cellular forms: soluble and insoluble. The former mostly exists in the cytoplasm, whereas the latter attaches to the cell membrane. The distribution of these two forms is regulated by the phosphorylation of sucrose synthase. Sucrose phosphate synthase is one of the key enzymes influencing sucrose metabolism; it irreversibly catalyzes fructose 6-phosphate and uridine diphosphate glucose to synthesize sucrose 6-phosphate, which then generates sucrose under the action of sucrose phosphatase (Lutfiyya et al., 2007). Fructose transformed from sucrose in the sink tissue must undergo phosphorylation before it can be further metabolized. Phosphofructokinase is the main enzyme that phosphorylates fructose (Schoneberg et al., 2013). It catalyzes the production of fructose 6-phosphate, which may influence starch synthesis. Transcription factors also regulate carbohydrate metabolism. The NAC transcription factor *CINAC68* positively regulates sugar content and seed development in watermelons by repressing *CIINV* and *CIGH3.6* (Wang et al., 2021). *PuWRKY31* binds to the *PuSWEET15* promoter and induces its transcription to increase sugar content in the fruit of the “Nanguo” bud sport (Li et al., 2020).

Sugar content is a quantitative trait, and the number of QTLs related to sugar content during fruit ripening and the corresponding genes remain uncertain. In this study, our objectives were to identify QTLs associated with total sugar content by SLAF-seq and to identify candidate genes within the QTL-related regions by RNA-seq. These research results may lead to a breakthrough in the molecular mechanism of carbohydrate metabolism and will have important practical implications for guiding the breeding of sweet pear cultivars.

## Material and methods

### Plant materials

A sweet cultivar of “Zaoshengxinshui,” a general cultivar of “Qiushui” and their crossing progenies (92 individuals), was investigated in this study. “ZQ65” (sweet) and “ZQ82” (not sweet) were selected from the hybrid progenies. All accessions belonged to *Pyrus pyrifolia* and were planted on the experimental farm of the Shanghai Academy of Agricultural Sciences in Zhuanhang Town (Shanghai, China). The fruits were sampled at 90 and 100 days after blossom (DAB) in 2017 and 2018, respectively, for QTL analysis. The fruits of “Zaoshengxinshui,” “Qiushui,” “ZQ65,” and “ZQ82” were sampled at 75, 82, 89, 96, 103, and 110 DAB, respectively, in 2020 for RNA-seq analysis. Five fruits were collected from each sample in biological replicates. Three biological replicates were used for each experiment.

### Evaluation of sugar contents

Four individual sugars (sucrose, fructose, glucose, and sorbitol) were tested in the fruits of all samples using high-performance liquid chromatography. The pear flesh was squeezed and centrifuged at 10,000 rpm for 10 min. The supernatant (100 μl) was aspirated and diluted 10 times. The filtrate was collected through a 0.22-μm filter membrane to determine soluble sugar contents. Chromatographic conditions were determined as a mobile phase: ultra-pure water; flowrate, 1.0 ml/min; column temperature, 80°C; injection volume, 10 μl; liquid chromatography sugar column (SP0810), 8×300 mm (Shodex, Japan); and differential refractive index detector, Waters 2414 (Waters, USA).

### QTL mapping

A linkage map was constructed based on our previous research (Jiang et al., 2022). The SLAF markers and correct genotyping errors were ordered within the LGs using the HighMap software. MapQTL6 was used for interval mapping of quantitative trait loci for two individual years (2017 and 2018) (Van Ooijen, 2009). A single-linkage clustering algorithm was used to cluster the markers into linkage groups (LGs) based on the independence logarithm of odds (LOD) score as a distance metric. The significance threshold for the LOD score was set at 3.

### RNA extraction and RNA-Seq

The fruits of “Zaoshengxinshui,” “Qiushui,” “ZQ65,” and “ZQ82” were subjected to RNA extraction. At each time point, the flesh of the five fruits was collected for biological replication. “Zaoshengxinshui” and “Qiushui” had three biological replicates. In each of “ZQ65” and “ZQ82,” three biological replications at each time points were collected and pooled equally into one sample for RNA-seq. Forty-eight samples were tested. The cetyltrimethyl ammonium bromide (CTAB) method was used to extract total RNA from the fruit flesh. RNA concentration and purity were evaluated using a NanoDrop 2000 spectrophotometer (Thermo Fisher Scientific, USA). Genomic DNA was digested with DNase I, and cDNA libraries were constructed using the NEBNext Ultra RNA Library Prep Kit (NEB Inc., USA) according to the manufacturer’s instructions. Library quality was evaluated using an Agilent Biological Analyzer 2100 system. Paired-end sequencing was performed using the Illumina HiSeq 4000 system (Illumina, USA). Pre-processing filters and trims of raw data were used to remove reads containing adapters, reads of more than 5% of unknown nucleotides, and low-quality reads containing more than 20% bases of quality value ≤ 10. Only clean reads were used in the subsequent analyses.

### Identification of significantly differentially expressed genes

The HISAT2 software (Kim et al., 2015) compares the filtered reads to the reference genome of “Dangshansuli” (Wu et al., 2013). We used HTSeq statistics to compare the read count value of each gene with the original expression level of the gene. The read count was positively correlated with the true expression level, the length, and the sequencing depth of the genes. To ensure that the gene expression levels between different genes and different samples were comparable, we used fragment per kb per million reads (FPKM) to standardize the expression level. DESeq was used to perform a differential gene expression analysis. The base mean was calculated to represent the homogenization result of the gene read count of all samples of the two groups for comparison. The threshold to determine the significance of the gene expression difference was set to |log2FoldChange(baseMean)| > 1 and a significance *p-*value < 0.05. Kyoto Encyclopedia of Genes and Genomes (KEGG) pathway analysis was performed by first mapping all DEGs to KEGG terms in the database (https://www.genome.jp/kegg/pathway.html) and calculating the gene numbers for every term to identify significantly enriched KEGG terms in the input list of DEGs. A heatmap of the gene expression in each module was obtained using MEV 4.0.

### Weighted gene co-expression network analysis

WGCNA was used to create a cluster of genes with similar expression modes and to analyze the relationship between modules and sugar content. The FPKM values for the QTL-related genes were sorted. Reflecting the excessive number of genes, the WGCNA package was run in the R language under insufficient memory. According to the computer configuration, the genes with low FPKM values <0.1 in 40% of samples were removed.

### Expression analysis of quantitative real-time PCR

Four pear accessions (“Zaoshengxinshui,” “Qiushui,” “ZQ65,” and “ZQ82”) at six time points were used to extract RNA. At each time point, all accessions had three biological replications. A total of 72 samples were analyzed. First-strand cDNA was synthesized using the PrimerScript RT Reagent Kit with gDNA Eraser (RR047, Takara, Osaka, Japan). The real-time PCR mixture (10 μl total volume) contained 5 μl of TB Green Premix Ex Taq (RR420A, Takara, Osaka, Japan), 0.5 μl of each primer (10 μM), 0.5 μl of cDNA, and 3.5 μl of double-distilled water. The reactions were performed on a LightCycler 480 instrument (Roche, Basel, Switzerland), according to the manufacturer’s instructions. The two-step quantitative PCR (qPCR) program was initiated at 95°C for 30 s, followed by 40 cycles of 95°C for 5 s and 60°C for 30 s. The expression was calculated as 2^−ΔΔCt^ and normalized to the expressions of the actin gene (JN684184) and UBI (XM_009368893.2). All the primers used are listed in **Supplementary Table S1**.

## Results

### Sugar content in the fruits of “Zaoshengxinshui,” “Qiushui,” and their progenies

**Figure 1:**
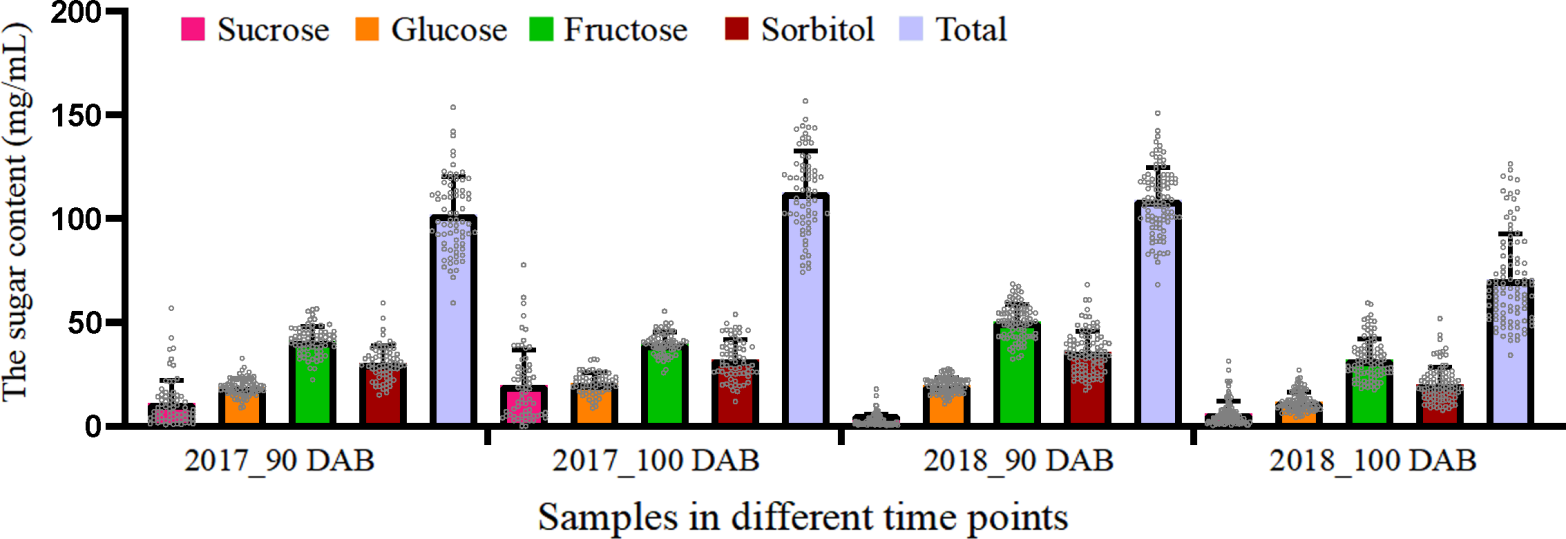
Average content of four types of sugars in “Zaoshengxinshui” and “Qiushui” and their 92 offspring.

The contents of the four types of sugars were measured in the “Zaoshengxinshui,” “Qiushui,” and 92 progenies. Fructose accounted for the largest proportion of all sugars (**Figure 1**), ranging from 35.4% to 45.7%. Sorbitol accounted for the second highest proportion, and the ratio ranged from 28.5% to 32.8%. The content of sucrose was the lowest (ratio, 2.7% to 17.5%). The total sugar content varied widely in all the samples. The highest numbers were 156.76 mg/ml at 100 DAB in 2017 and 150.94 mg/ml at 90 DAB in 2018. The lowest numbers were 74.30 mg/ml at 100 DAB in 2017 and 68.31 mg/ml at 90 DAB in 2018. The total sugar content increased from 90 to 100 DAB in 2017 but decreased in 2018. These findings demonstrate that although the total sugar content always increases during fruit ripening, it may also decrease in a short time.

### QTL mapping

**Figure 2:**
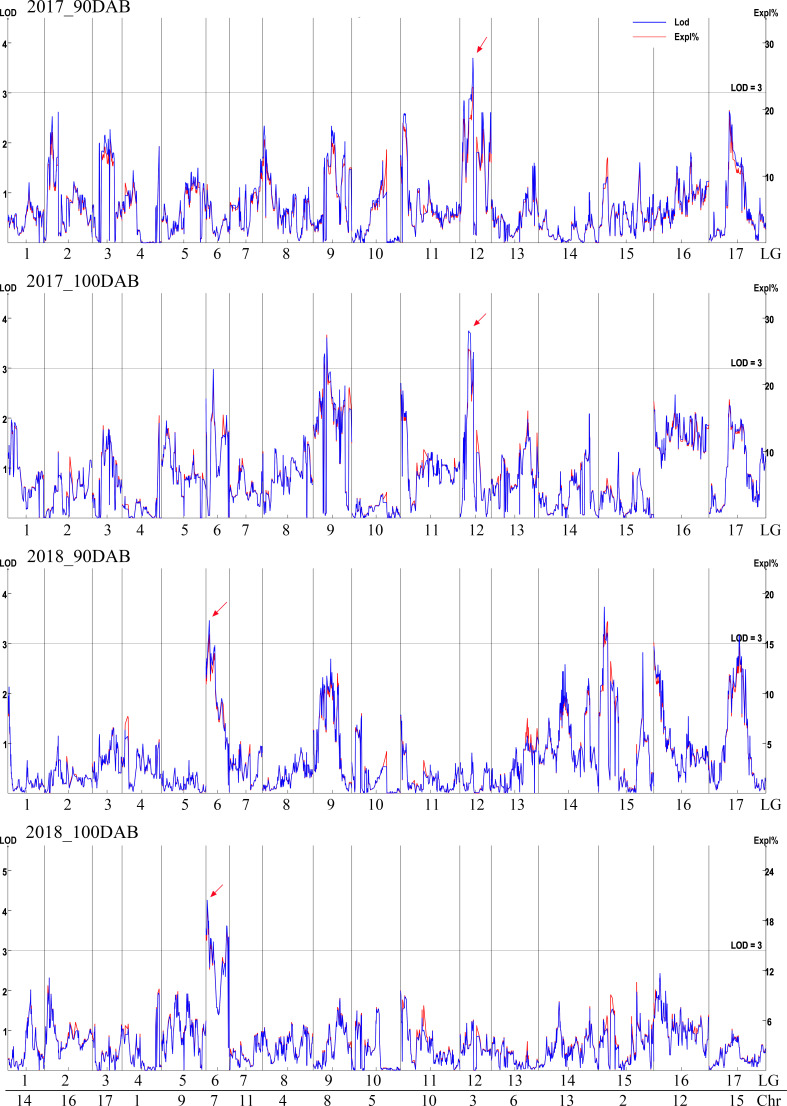
QTL analysis of total sugar content in 2017 and 2018. The logarithm of odds (LOD) value is shown on the left y-axis, and the explained variance (Expl %) is on the right y-axis.

The genetic map was based on our previous study (Jiang et al., 2022). Interval mapping was used to identify putative QTLs for total sugar content-related traits at 90 and 100 DAB in two independent years (2017 and 2018). The LOD value was calculated for each SLAF tag (**Figure 2**). Although the data for the same year differed, fluctuations occurred in the LOD value of every tag; the same peaks were observed. In 2017, the highest LOD numbers were 3.70 and 3.75 at 90 and 100 DAB, respectively. An obvious peak on the LOD and explained variation values graph was observed at 42.212 cM in LG12, which had the highest average LOD number (3.44). In 2018, the highest number of LOD was 3.73 and 4.26 at 90 and 100 DAB, respectively. The position of 8.879 cM in LG6 had the highest average LOD (3.45) at 90 and 100 DAB. Two sugar-related QTLs (qSugar-LG12-Chr3 and qSugar-LG6-Chr7) were identified.

### QTL-region-associated genes

A total of 577 genes around the QTL locus of qSugar-LG6-Chr7 were isolated, and 119 genes were annotated using KEGG pathways. Eight genes were enriched for starch and sucrose metabolism (**Supplementary Figure S1**). Eight genes were enriched in fructose and mannose metabolism, including six genes of *sorbitol dehydrogenase*. Five *ethylene-responsive transcription factors*, a *soluble acid invertase* gene, and a gene of *starch synthase 1* were found near this locus. In the chromosome region around qSugar-LG12-Chr3, 519 genes were isolated and 90 genes were annotated by KEGG pathways. One gene was enriched in fructose and mannose metabolism, and two genes were enriched in starch and sucrose metabolism (**Supplementary Figure S2**). These genes were annotated as *alpha-glucan phosphorylase*, *beta-glucosidase*, and *6-phosphofructo-2-kinase*, respectively. The genes related to QTLs were putative trigger genes that increased sugar content during pear fruit ripening.

### Sugar content in fruit ripening and RNA-seq analysis

**Figure 3:**
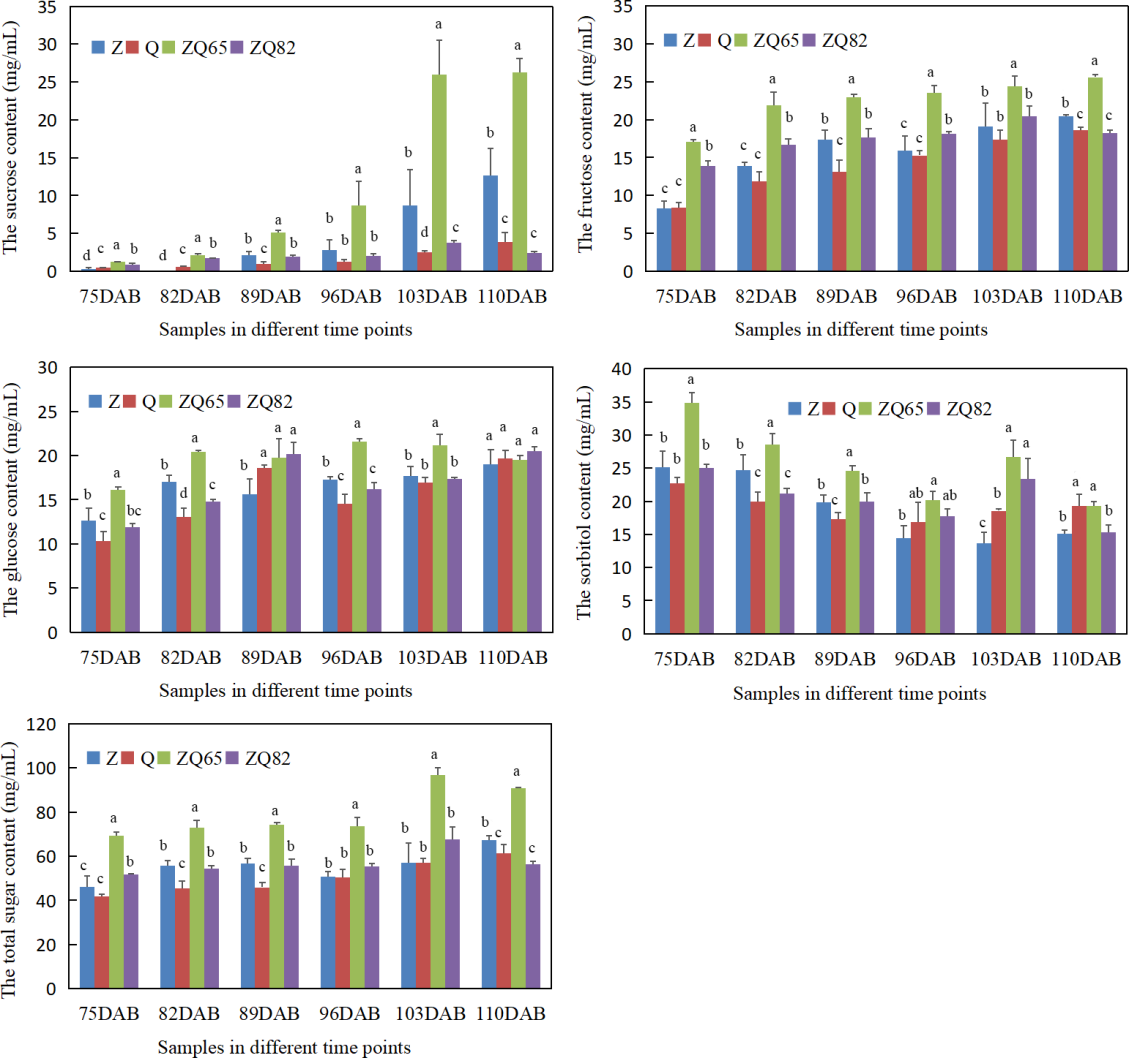
Sucrose, fructose, glucose, sorbitol, and total sugar contents in four cultivars during fruit ripening. Statistical analyses were performed at each time point using the analysis of variance (ANOVA) and Duncan tests, Different low case letters above columns indicate statistical differences at *p* ≤ 0.05.

Four individual sugars (sucrose, fructose, glucose, and sorbitol) were tested from 75 to 110 DAB prior to maturation. All individual and total sugar contents increased during fruit ripening (**Figure 3**). The sucrose content increased in all four cultivars. “Zaoshengxisnhui” and “ZQ65” had the highest sucrose contents at 103 and 110 DAB. For fructose, “ZQ65” had the highest content at all time points. “Zaoshengxinshui” had the second highest content at 110 DAB. Although glucose contents differed in all samples at the first five time points, it was not significantly different at 110 DAB. The sorbitol content showed an interesting down–up–down trend. A high content of sorbitol was found in “ZQ65” and “ZQ82” at 103 DAB, which decreased at 110 DAB. The highest total sugar content was observed in “ZQ65” at all time points, and “Qiushui” had the lowest total sugar content at most time points (**Figure 3**). Overall, three phenomena were observed. In sweet cultivars, sucrose and fructose accumulated significantly, and the sorbitol content decreases significantly during fruit ripening.

Four samples (“Zaoshengxinshui,” “Qiushui,” “ZQ65,” and “ZQ82”) at six time points were evaluated *via* transcriptome analysis. After removing low-quality sequences and adapters, a total of 2.05 billion clean paired-end reads were obtained (**Supplementary Table S2**). The clean reads for each sample ranged from 5.7 to 7.4 GB, and the Q30 value was ≥91.21% (**Supplementary Table S2**). All reads for each sample were aligned with the reference genome of “Dangshansuli,” and the efficiency of the alignment ranged from 72.60% to 80.46%.

### Identification and annotation of differentially expressed genes

**Figure 4:**
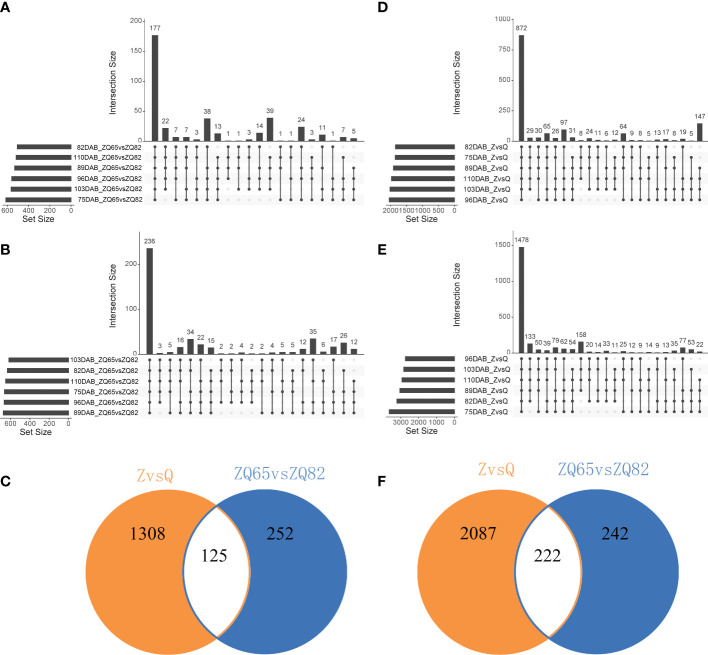
The number of DEGs in “Zaoshengxinshui” **(Z)**, “Qiushui” **(Q)**, “ZQ65,” and “ZQ82” at six time points. **(A)** Upregulated DEGs in “ZQ65” vs. “ZQ82.” The upright column represents the number of DEGs at different time points, and the lower dot indicates the time points. The left horizontal column represents the number of DEGs at a single time point. **(B)** Upregulated DEGs in “Zaoshengxinshui” vs. “Qiushui.” **(C)** The intersection of upregulated DEGs in both “ZQ65” vs. “ZQ82” and “Zaoshengxinshui” vs. “Qiushui.” **(D)** Downregulated DEGs in “ZQ65” vs. “ZQ82.” **(E)** Downregulated DEGs in “Zaoshengxinshui” vs. “Qiushui.” **(F)** The intersection of downregulated DEGs in both “ZQ65” vs. “ZQ82” and “Zaoshengxinshui” vs. “Qiushui.”.

**Figure 5:**
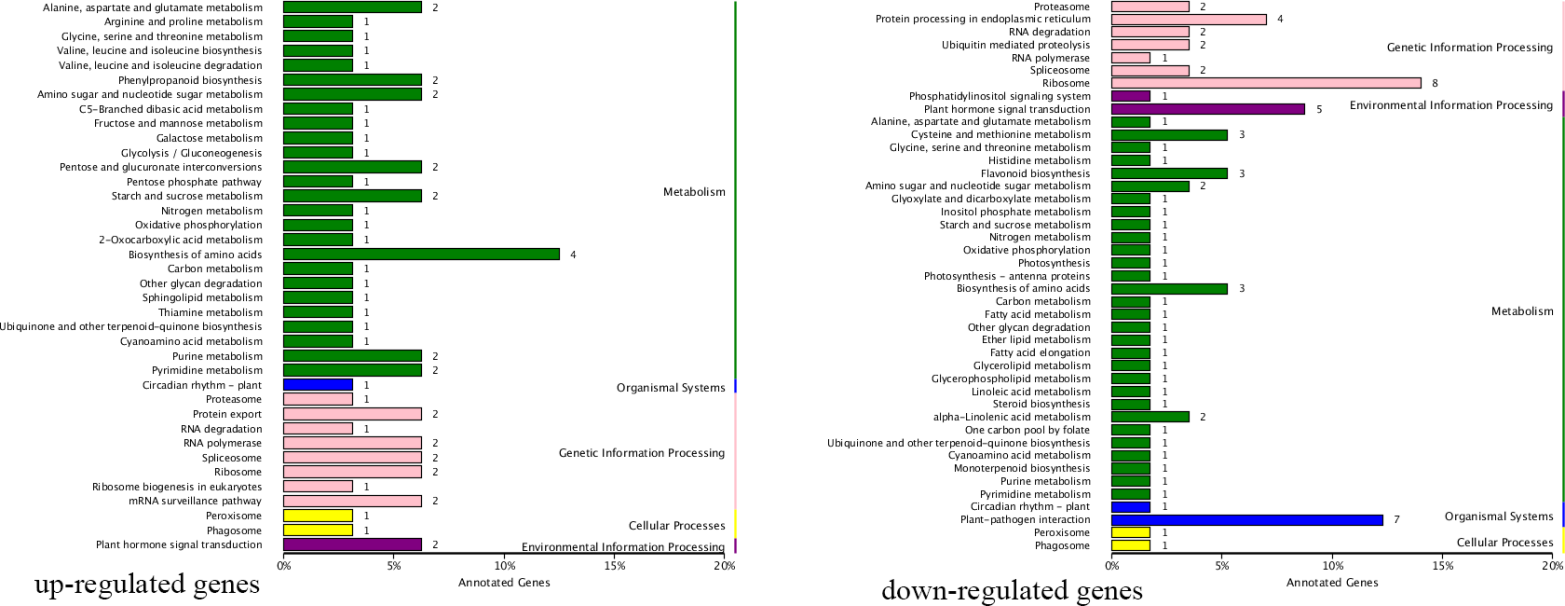
Differentially expressed genes enriched in certain KEGG pathways. The number next to each bar represents the number of enriched genes.

Parents and progenies were compared separately. A total of 8,988 DEGs and 2,573 DEGs were identified in pairwise comparisons (“Zaoshengxinshui” vs. “Qiushui” and “ZQ65” vs. “ZQ82” at six time points) (**Figure 4**). In “ZQ65” vs. “ZQ82,” 177 upregulated and 872 upregulated DEGs were noted at all time points (**Figures 4A, B**). In “Zaoshengxinshui” vs. “Qiushui,” 872 upregulated and 1,478 upregulated DEGs were observed at all time points (**Figures 4D, E**). Overall, 125 upregulated and 222 downregulated DEGs were common in all comparisons (**Figures 4C, F**). Seven genes were selected for validation using qPCR. The differential expression trends detected using qPCR and RNA-seq were consistent (**Supplementary Figure S3**), indicating the reliability of the RNA-seq results. KEGG pathway analysis showed that several DEGs were enriched in some pathways (**Figure 5**). Among the 125 upregulated genes, one gene was enriched in fructose and mannose metabolism, two genes were enriched in starch and sucrose metabolism, and two genes were enriched in plant hormone signal transduction. Of the 222 downregulated genes, one gene was enriched in starch and sucrose metabolism, five genes were enriched in plant hormone signal transduction, and two genes were enriched in amino sugar and nucleotide sugar metabolism.

### Differentially expressed genes related to sugar, hormones, and transcription factors

**Figure 6:**
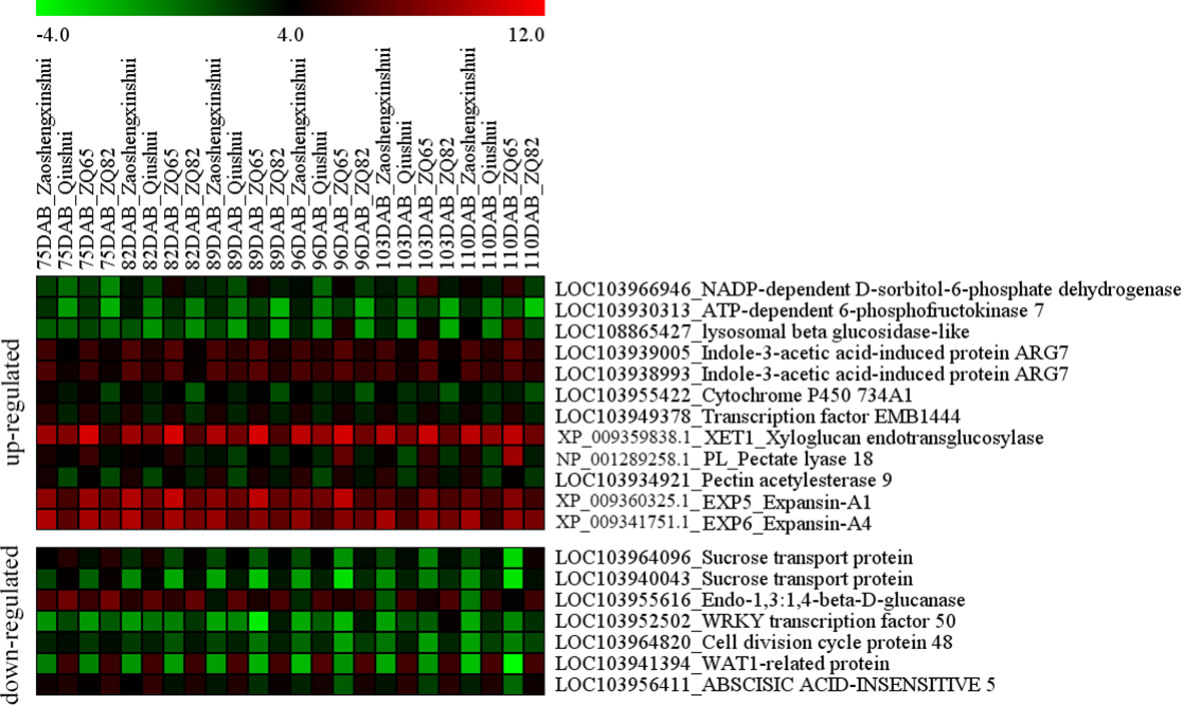
Heatmap of the differentially expressed genes in the fruit ripening of four pear cultivars (“Zaoshengxinshui,” “Qiushui,” “ZQ65,” and “ZQ82”).

The intersection of DEGs related to sugar, hormones, and transcription factors among “ZQ65” vs. “ZQ82” and “Zaoshengxinshui” vs. “Qiushui” were analyzed (**Figure 6**). Among the upregulated genes in the sweet cultivars, one gene encoding *ATP-dependent 6-phosphofructokinase 7* (*PpPFK*, LOC103930313) and one gene encoding *NADP-dependent D-sorbitol-6-phosphate dehydrogenase* (*PpS6PDH*, LOC103966946) were identified. These two genes influence the metabolism of fructose and sorbitol. A *beta-glucosidase* gene (LOC108865427) was upregulated in the “Zaoshengxinshui” and “ZQ65,” which was related to cellulose decomposition. Two genes, *indole-3-acetic acid-induced protein PpARG7* (LOC103939005 and LOC103938993), and two transcription factors (*PpEMB1444* and LOC103949378 and *PpCYP734A1* and LOC103955422) were identified. Three genes (*Xyloglucan endotransglucosylase*, XP_009359838.1; *Pectate lyase 18*, NP_001289258.1; and *Pectin acetylesterase 9*, LOC103934921) were related to pectin and xyloglucan, which were all upregulated in the sweet cultivars. Two *expansin* genes (*PpEXP5*, XP_009360325.1 and *PpEXP6*, XP_009341751.1) were also upregulated. The upregulation of these genes suggested that they were closely related to sugar content and fruit ripening. Two *sucrose transport genes* (*PpSUT*, LOC103964096, and LOC103940043) were isolated. These two genes were highly similar and identified as different members of the same gene. The expression of these two genes was negatively correlated with sugar content. An *endo-1,3:1,4-beta-D-glucanase* gene (LOC103955616) that influences the decomposition of plant fibers was identified. Two transcription factors (*PpWRKY50*, LOC103952502, and *abscisic acid-insensitive 5*, LOC103956411) were downregulated in sweet cultivars. The *cell division cycle gene 48* (LOC103964820) and *WAT1-related gene* (LOC103941394) were both downregulated in the sweet cultivars. These genes were negatively correlated with the sugar content and fruit ripening.

### QTL-related regions associated gene expression

A total of 577 genes related to qSugar-LG6-Chr7 and 519 genes related to qSugar-LG12-Chr3 (1,096 genes in total) were analyzed for their expression level. We used two methods to screen for the target genes. First, the genes expressed differently in both “Zaoshengxinshui” vs. “Qiuhsui” and “ZQ65” vs. “ZQ82” at more than four time points were isolated (**Supplementary Figure S4**, **Supplementary Table S3**). In the chromosomal region related to qSugar-LG6-Chr7, four genes were upregulated in the sweet cultivars, including a gene encoding *pectin acetylesterase 9* (LOC103934921) and a gene encoding *CDPK-related kinase 4* (LOC103951600). Four downregulated genes were identified in the sweet cultivars, including a gene encoding of *4-coumarate-CoA ligase 2* (LOC103951504) and a gene of *exordium-like* (LOC103959847). In the chromosome region related to qSugar-LG12-Chr3, 10 genes were upregulated in the sweet cultivars, including putative *E3 ubiquitin-protein ligase XBAT31* (LOC103935753), *Clathrin interactor 1* (LOC103933447), and *CAX-interacting gene 4* (LOC103954326). Among the downregulated genes in the sweet cultivars, eight genes were identified, including *mitogen-activated protein kinase 3* (LOC103948905), *protein accelerated cell death 6* (LOC103933437), and *probable serine/threonine-protein kinase PBL9* (LOC103954305). These genes represented putative key genes related to QTL loci.

**Figure 7:**
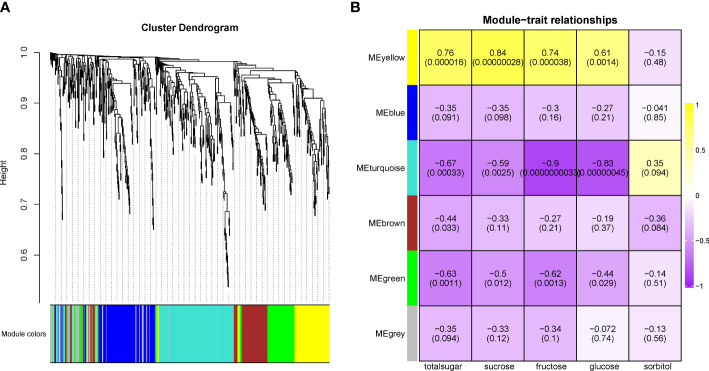
Weighted gene co-expression network analysis in genes related to QTL loci. **(A)** The cluster dendrogram in 649 genes. **(B)** The relationship between modules and traits.

**Figure 8:**
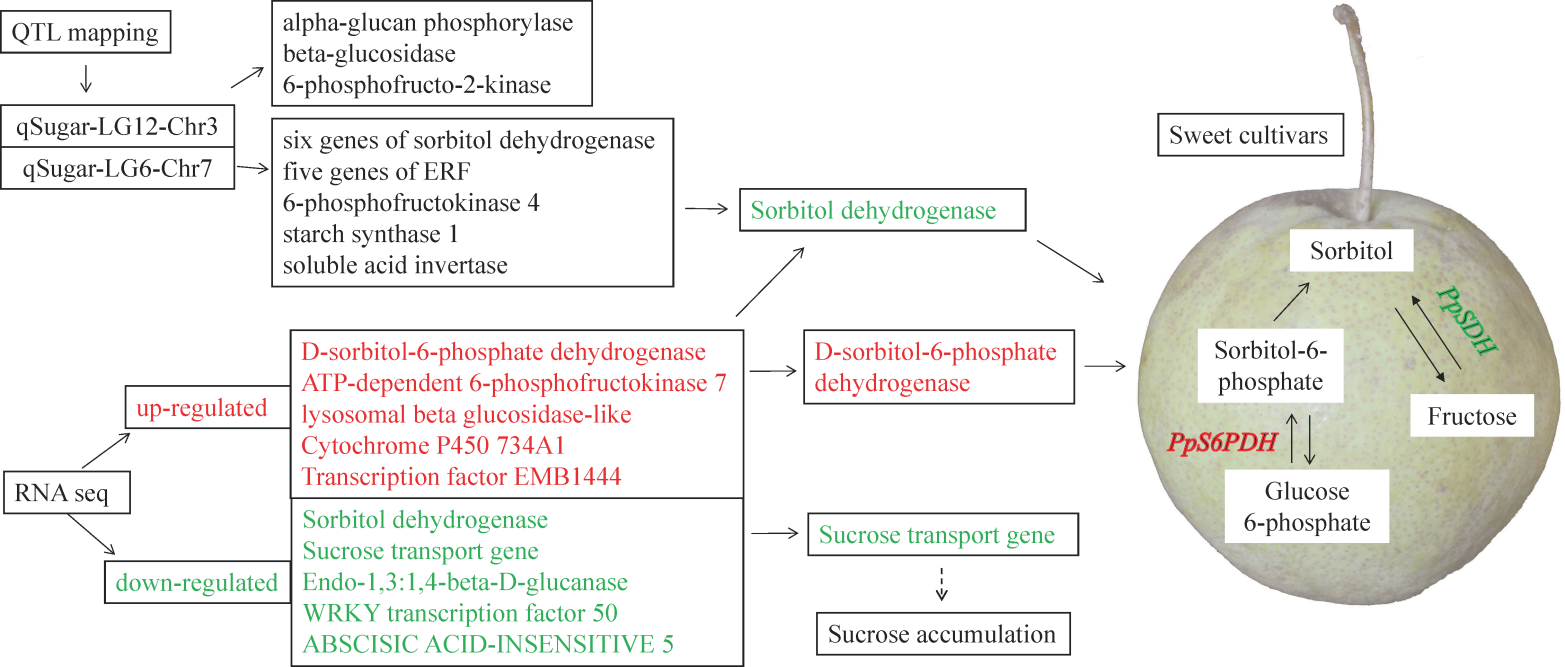
Putative regulation network of the sugar content in the sweet cultivars with high content of fructose. The upregulated genes in the sweet cultivars are labeled in red. The downregulated genes are labeled in green.

Second, the WGCNA analysis was used to isolate putative key genes from 1,096 genes that were QTL related. A total of 447 genes were removed based on a low FPKM value (<0.1) in more than 40% of the samples. The remaining 649 genes were clustered into six modules (**Figure 7A**, **Supplementary Table S4**). The MEyellow module was positively related to the contents of total sugar, sucrose, fructose, and glucose but negatively related to the sorbitol content (**Figure 7B**). The remaining five modules were all negatively related to the total sugar content, with MEturquoise being the most negative module. Three genes related to sugar signals were found in the MEyellow module. Three genes, namely, *sugar kinase slr0537* (LOC103951483), *ATP-dependent 6-phosphofructokinase 4* (LOC103952760), and *starch synthase 1* (LOC103948046), were upregulated in the sweet cultivars (“Zaoshengxinshui” and “ZQ65”). The gene encoding the auxin-responsive gene *SAUR32* (LOC103951601) was also upregulated in sweet cultivars. Two genes, sorbitol dehydrogenase (*PpSDH*, LOC103960512 and LOC103960513), were observed in the MEturquoise module. Their expression was negatively related to fructose content, which was downregulated in the sweet cultivars. The *Dof zinc finger protein DOF3.7* (LOC103951625) gene and a gene encoding *ethylene-responsive transcription factor CRF4* (LOC103958171) were downregulated in the sweet cultivars. These genes were negatively co-expressed with fructose and glucose. We identified a putative regulatory network for sugar content (**Figure 8**). The genes of *PpSDH* and *PpSUT* might help regulate sugar content during fruit ripening.

## Discussion

Multiple chemicals are involved in pear fruit ripening. This study focused on sugar accumulation, which is an important factor in fruit quality. In the “Yali” pear (*P. bretschneideri*), fructose was the dominant sugar, followed by glucose and sucrose (Chen et al., 2006). In “Hosui” (*P. pyrifolia*), “Kuerlexiangli” (*P. sinkiangensis*), “Nanguoli” (*P. ussuriensis*), and “Starkrimson” (*P. communis*), fructose also accounts for the greatest sugar content at maturity from components of sucrose, glucose, fructose, and sorbitol (Zhang et al., 2016). Fructose is the dominant sugar in apples (Karadeniz and Ekşi, 2002). In this study, sucrose and fructose significantly accumulated during fruit ripening in “Zaoshengxinshui,” “Qiushui,” and their progenies, and these two sugars accounted for the highest sugar content (**Figure 3**). This finding implies that the accumulation of fructose and sucrose is a key factor in sweet pears. The sorbitol content was not significantly changed in fruit ripening of plums (Usenik et al., 2008), and it has fluctuated in different periods during the developmental period for the fruit of pear “Dangshansuli” (Yu et al., 2019). In the sweet pear “Zaoshengxinshui” and “ZQ65,” a decrease in sorbitol content was observed, which suggested that the degradation of sorbitol may influence sweet pears.

The sugar content is related to several QTLs. In pears, QTLs for soluble solid content were identified in LG5 and LG15 (Qin et al., 2022). Two QTLs were located in LG 1 and LG 7 based on sucrose, fructose, and glucose contents in Japanese pears (Nishio et al., 2018). In a genome-wide association study, an SNP located on chromosome 7 and an SNP on chromosome 11 strongly influenced sucrose and glucose accumulations, respectively (Nishio et al., 2021). The LG7/chromosome 7 was commonly found in these two reports. In this study, a QTL associated with total sugar content was also identified on chromosome 7, and the average LOD value was 3.45 in 2 years (**Figure 2**). This locus (qSugar-LG6-Chr7) was near the locus of Chr07_33139082 in a study by Nishio et al. (2021). “Zaoshengxinshui” was the offspring of Japanese pear “Shinsui”. The close genetic background implies that the sugar content-related QTLs were similar in some Chinese and Japanese pears. A newly identified locus of qSugar-LG12-Chr3 was identified by the sugar content in this study. QTLs on chromosome 3 were mostly related to harvesting dates in several studies (Kunihisa et al., 2014). The sugar content was positively correlated with the harvesting date, and the fruits of early maturing cultivars became sweet earlier. The pear has exhibited gametophytic self-incompatibility. QTL localization can only be performed using the F1 generation and the large annual variation in traits, which limits the study of pear trait marker associations. Additional QTL localization studies using several F1 generations could identify common loci.

RNA-seq analysis was used to identify DEGs among sweet and general cultivars. For usual sorbitol producers, sorbitol-6-phosphate dehydrogenase (S6PDH) and sorbitol-6-phosphate phosphatase (S6PP) are the key synthetic enzymes (Pleyerova et al., 2022). S6PDH is a key enzyme that regulates partitioning between sorbitol and sucrose in apple leaves, and an overexpression of *S6PDH* increases sorbitol and sucrose contents (Kanamaru et al., 2004). In this study, *PpS6PDH* was upregulated in the fruit of sweet cultivars. Considering that *PpS6PDH* is a reversible enzyme, its role in fruit ripening requires further investigation. In apples, activation of a *pyrophosphate-dependent phosphofructokinase* gene can promote soluble sugar accumulation (Yu et al., 2022). In sweet pear cultivars evaluated in the present study, the gene encoding *6-phosphofructokinase 7* was upregulated. This implies that this gene influences the regulation of the sugar content. Two *sucrose transporter* genes (*PpSUT*) were downregulated in sweet cultivars. *SUT* strongly influences phloem loading and unloading processes and efflux sucrose into source or sink apoplasms (Kuhn and Grof, 2010). An overexpression of a grapevine *sucrose transporter* (*VvSUT27*) in tobacco showed that the total sugar contents in the roots and stems of transformants were higher than those in the control plants. No significant difference was observed in the leaves between the transformants and control plants (Cai et al., 2017). During fruit ripening, the sugar content in the pear fruits was higher than that in the leaves. Downregulating *PpSUT1* may prevent the outflow of sugar from fruits and promote sugar accumulation in fruits. Except for sugar-related genes, *indole-3-acetic acid-induced protein ARG7* was upregulated in sweet cultivars. This gene is induced by IAA and is associated with the fruit flesh softening rate in peaches (Carrasco-Valenzuela et al., 2019). This upregulation might be related to the sugar content. Five genes were related to the cell wall, such as *XTH* and *expansin*. XTH is a cell-wall-relaxant enzyme involved in plant cell wall remodeling (Li et al., 2021). Expansins are plant cell-wall-loosening proteins (Sampedro and Cosgrove, 2005). The upregulation of these genes implied that the cell wall was loosened, inducing fruit softening in the sweet cultivars. Cell wall loosening and increase in sugar content are key factors in fruit ripening.

Our study identified two QTLs associated with sugar content. The genes around these two loci might be the trigger genes that regulate the sugar content of individuals. qSugar-LG6-Chr7 was identified in another report (Nishio et al., 2021) and our study. In Nishio’s study, the acid invertase gene *PPAIV3* strongly affected sucrose and glucose accumulation. We also found that *PPAIV3* was near the locus of qSugar-LG6-Chr7, but transcriptome analysis showed that the expression of *PpAIV3* was low, and some samples could not detect its expression at all. Therefore, *PpAIV3* may not be a key gene. Interestingly, six genes of *SDH* and five genes of *ERF* were located around this locus. Six *SDH* genes were clustered together. In the *Pyrus pyrifolia* “Cuiguan” genome, 12 *SDH* genes were observed, with half on chromosome 1 and half on chromosome 7. On chromosome 1, five *SDH* genes clustered together, and only one gene was scattered. A whole-genome or large-fragment duplication occurring in the pear genome may explain why chromosomes 1 and 7 have duplicate copy regions (Xiang et al., 2017). The aggregation of *SDH* genes on chromosomes requires further investigation. *SDH* strongly influences the degradation of sorbitol in plants (Pleyerova et al., 2022), as it catalyzes a reversible reaction between D-sorbitol and D-glucose/D-fructose. In this study, two *SDH* genes around the qSugar-LG6-Chr7 locus were downregulated in sweet cultivars. In the pear fruit, downregulated *PpSDH* genes implied that the related pathway slowed down. In fructose-dominant cultivars, a decreased expression of this enzyme promoted fructose accumulation. *ERF* play a crucial role in regulating plant growth and developmental processes (Muller and Munne-Bosch, 2015). An ethylene response factor *TERF1* enhances glucose sensitivity in tobacco by activating the expression of sugar-related genes (Li et al., 2009). In this study, no difference in expression was observed in five genes of *ERF* around qSugar-LG6-Chr7, which suggested that *ERF* genes might not be involved in sugar regulation in pears.

In conclusion, this study identified sugar-associated QTLs and analyzed the expression of related genes using transcriptome analysis. Two QTLs (qSugar-LG12-Chr3 and qSugar-LG6-Chr7) were associated with the total sugar content. Several differential expressed genes related to fruit sugar accumulation were identified. *PpS6PDH* and *ATP-PpPFK* were upregulated, and the sucrose transporter *PpSUT* was downregulated in the sweet cultivars. WGCNA analysis showed that two *PpSDH* genes around the qSugar-LG6-Chr7 locus were negatively co-expressed with the total sugar content. This study helps to clarify sugar accumulation during pear maturity. Our new findings could provide theoretical guidance for precocious pear production and breeding.

## Data availability statement

The datasets presented in this study can be found in online repositories. The names of the repository/repositories and accession number(s) can be found below: https://ngdc.cncb.ac.cn/gsa/, PRJCA012773.

## Author contributions

SJ performed the experiments and wrote the manuscript. CS helped to estimate the traits. SL helped to analyze data and revise the manuscript. XW and JL involved in designing the research and revised the manuscript. All authors read and approved the manuscript.

## Supplementary material

Supplementary Figure 1 Annotation of 119 genes around the QTL locus of qSugar-LG6-Chr7 by KEGG pathways. The number next to each bar represents the number of genes.

Supplementary Figure 2 Annotation of 90 genes around the QTL locus of qSugar-LG12-Chr3 by KEGG pathways. The number next to each bar represents the number of genes.

Supplementary Figure 3 Expression of seven genes during pear fruit ripening. **(A)** Q-PCR validation of differential gene expression. **(B)** Linear regression analysis between the fold change of gene expression ratios obtained from RNA-seq and Q-PCR data based on Pearson correlation coefficient (R).

Supplementary Figure 4 Differently expressed genes in both ‘Zaoshengxinshui’ vs ‘Qiuhsui’ and ‘ZQ65’ vs ‘ZQ82’ around the QTL loci of qSugar-LG6-Chr7 and qSugar-LG12-Chr3.
