# Supplementary figures and images for: QTL mapping and transcriptome analysis of sugar content during fruit ripening of Pyrus pyrifolia
Source: Front Plant Sci. 2023 Mar 6;14:1137104. doi: 10.3389/fpls.2023.1137104 (PMC10025493; doi:10.3389/fpls.2023.1137104)

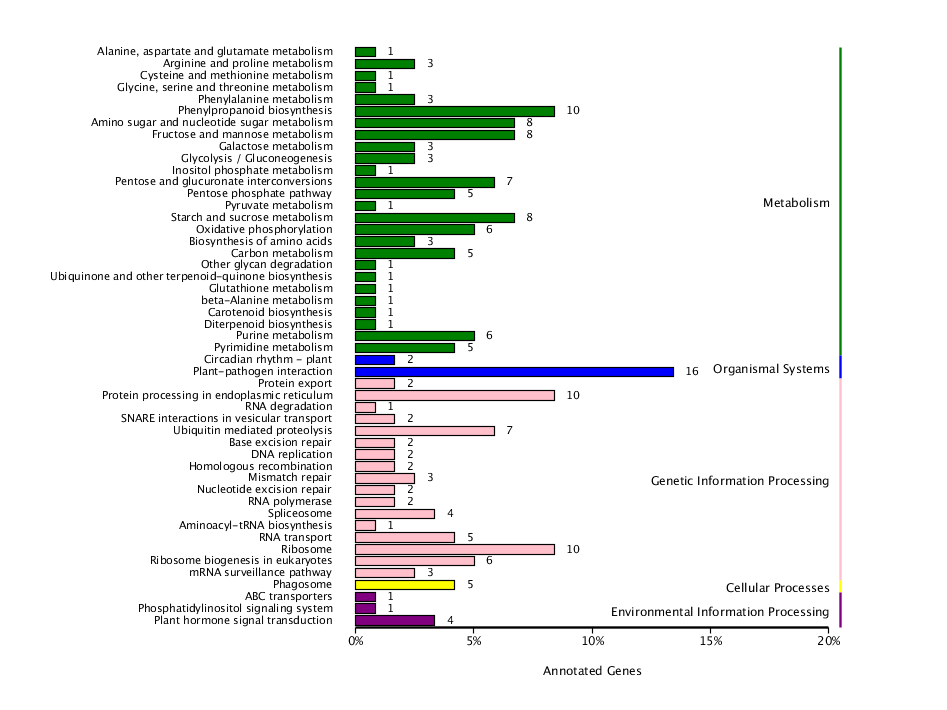

Supplement: Supplementary Figure 1 — Annotation of 119 genes around the QTL locus of qSugar-LG6-Chr7 by KEGG pathways. The number next to each bar represents the number of genes. [file Image_1.png]

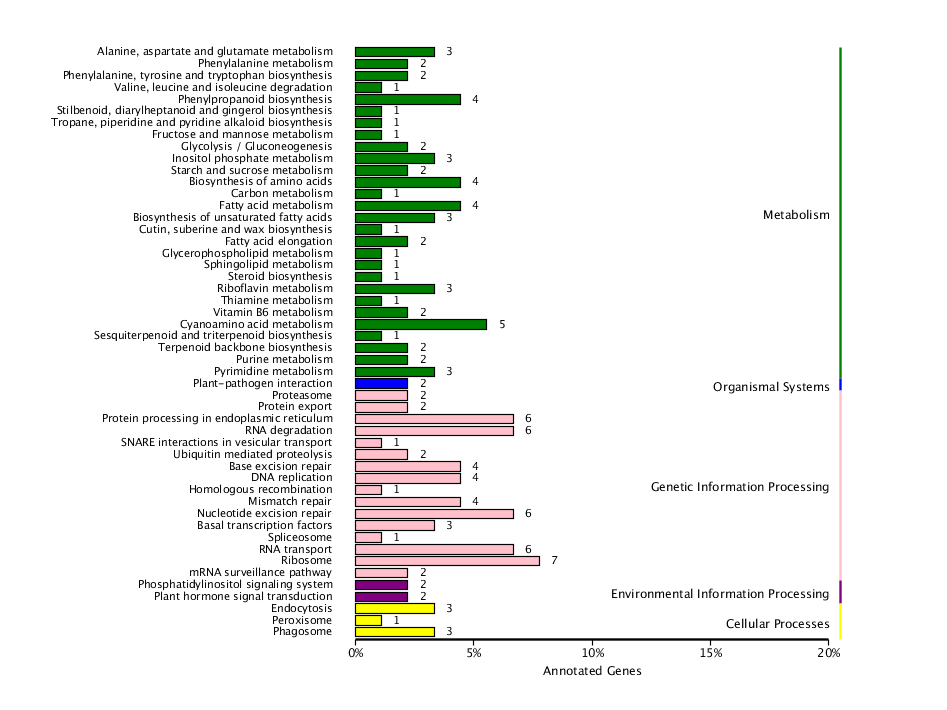

Supplement: Supplementary Figure 2 — Annotation of 90 genes around the QTL locus of qSugar-LG12-Chr3 by KEGG pathways. The number next to each bar represents the number of genes. [file Image_2.png]

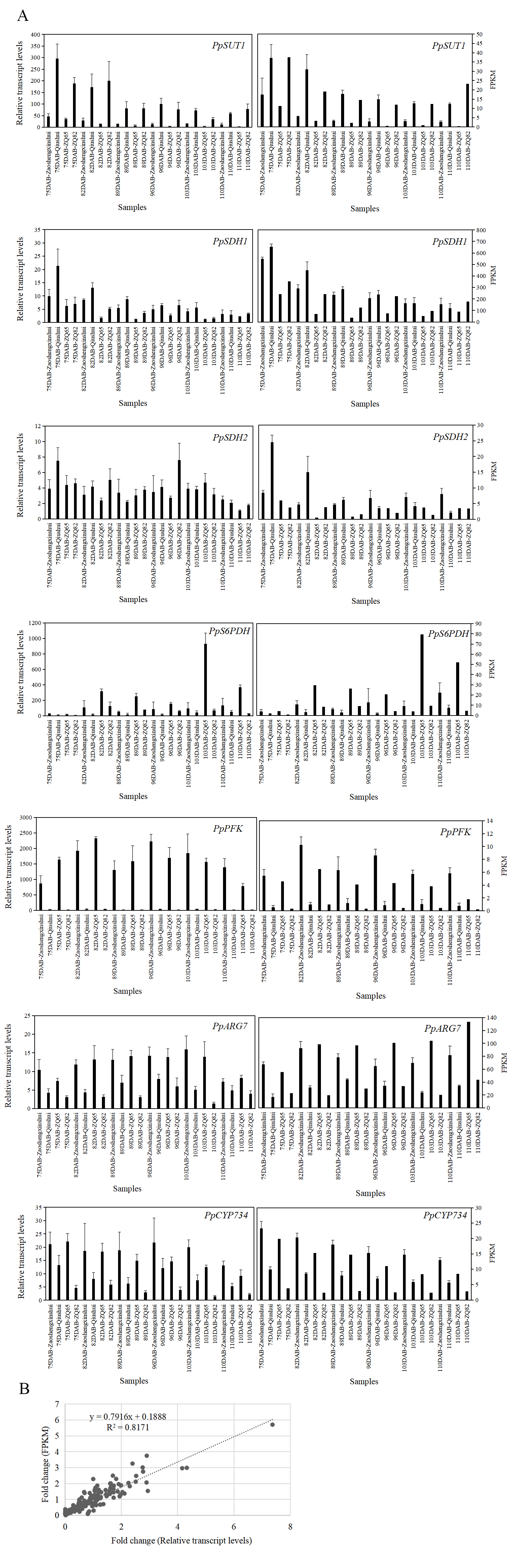

Supplement: Supplementary Figure 3 — Expression of seven genes during pear fruit ripening. (A) Q-PCR validation of differential gene expression. (B) Linear regression analysis between the fold change of gene expression ratios obtained from RNA-seq and Q-PCR data based on Pearson correlation coefficient (R). [file Image_3.tif]

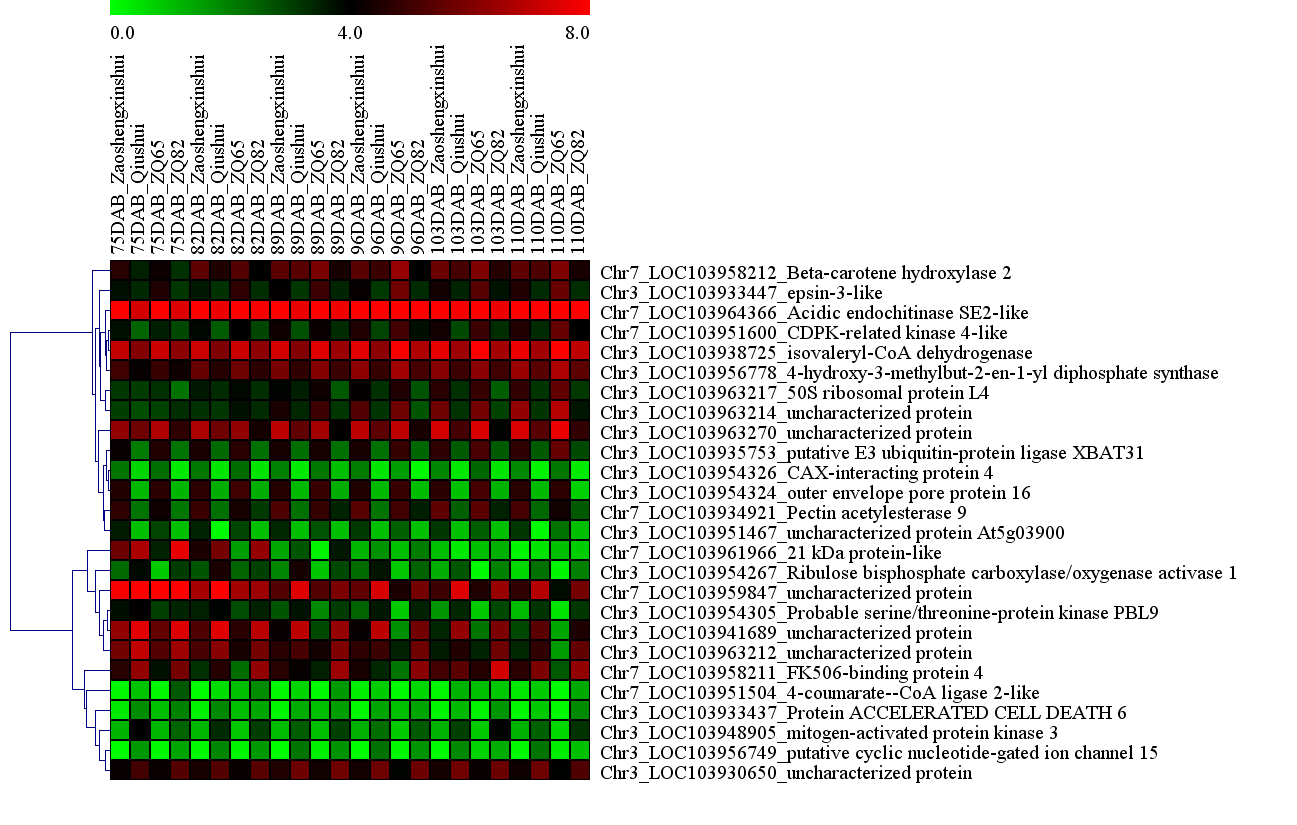

Supplement: Supplementary Figure 4 — Differently expressed genes in both ‘Zaoshengxinshui’ vs ‘Qiuhsui’ and ‘ZQ65’ vs ‘ZQ82’ around the QTL loci of qSugar-LG6-Chr7 and qSugar-LG12-Chr3. [file Image_4.tiff]
